# Near-Infrared Light-Controlled Dynamic Hydrogel for Modulating Mechanosensitive Ion Channels in 3-Dimensional Environment

**DOI:** 10.34133/bmr.0182

**Published:** 2025-04-09

**Authors:** Xiaoning Liu, Zimeng Zhang, Zhanshuo Cao, Hongbo Yuan, Chengfen Xing

**Affiliations:** ^1^ School of Materials Science and Engineering, Hebei University of Technology, Tianjin 300401, China.; ^2^ Key Laboratory of Molecular Biophysics of Hebei Province, School of Health Sciences and Biomedical Engineering, Hebei University of Technology, Tianjin 300401, China.; ^3^ School of Chemical Engineering, Hebei University of Technology, Tianjin 300401, China.; ^4^ Molecular Imaging and Photonics, Chemistry Department, KU Leuven, 3001 Heverlee, Belgium.

## Abstract

The extracellular matrix (ECM) creates a dynamic mechanical environment for cellular functions, continuously influencing cellular activities via the mechanotransduction pathway. Mechanosensitive ion channels, recently identified as key mechanotransducers, convert mechanical stimuli into electrical or chemical signals when they detect membrane deformation. This process facilitates extracellular Ca^2+^ influx, cytoskeletal reorganization, and transcriptional regulation, all of which are essential for cellular physiological functions. In this study, we developed a fibrous hydrogel composite (PIC/OEG-NPs) with near-infrared (NIR) light-controlled dynamic mechanical properties to modulate mechanosensitive ion channels in cells, by using oligo-ethylene glycol (OEG)-assembled polyisocyanide (PIC) polymer and OEG-grafted conjugated polymer nanoparticles (OEG-NPs). PIC and OEG-NPs assemble into PIC/OEG-NPs composites through OEG-mediated hydrophobic interactions when heated. Under NIR stimulation, the PIC/OEG-NPs composites exhibit increased mechanical tension and form tighter fibrous networks due to their thermoresponsive behavior. These changes are reversible and allow for the dynamic regulation of mechanosensitive ion channels, including Piezo1 in transfected HEK-293T cells and the endogenous TRPV4 in human umbilical vein endothelial cells (HUVECs), by switching NIR on and off. Furthermore, this process enhances the angiogenic potential of HUVECs. In summary, we present a simple and effective platform for in situ modulation of mechanosensitive ion channels in 3 dimensions.

## Introduction

Mechanical force serves as a crucial regulator of cell behavior and exerts an impact on various biological processes, including cell migration and stem cell differentiation [1,2]. Cells in biological tissues are located in a 3-dimensional (3D) environment filled with extracellular matrix (ECM), which provides a suitable mechanical environment for cell functions [3,4]. Cells perceive mechanical forces by converting them into biological signals that they can recognize, which is typically achieved through mechanotransduction pathway. Integrins are the principal cell adhesion transmembrane receptors. They function as connectors and sensors between the ECM and the cytoskeleton, and are involved in biochemical and mechanical signaling between cells and the environment during both normal tissue function and numerous disease states [5]. Cells transmit mechanical forces in the ECM environment through integrins, which promote focal adhesion kinase (FAK) phosphorylation and activate mechanical reactive signaling elements. At the same time, intracellular mechanical forces regulate the nuclear translocation of transcription regulatory factors YAP/TAZ, achieving the conversion of ECM mechanical signals into intracellular biological signals [6]. In the past few years, mechanosensitive ion channels (MSCs) have emerged as a key component of the cellular mechanotransduction system. MSCs sense mechanical forces on the cell membrane [7] and convert these stimuli into electrical or chemical signals, mediating extracellular Ca^2+^ influx, cytoskeletal rearrangement, and transcriptional regulation. This process plays a vital role in guiding normal dendritic branching, regulating central neuronal activity, and promoting wound healing [8,9]. Currently, 5 major categories of MSCs have been identified in eukaryotic organisms: the degenerate/epithelial sodium channel (DEG/EnaC), transient receptor potential (TRP) channels, 2-pore domain potassium channels (K2P), Piezo protein channels, and Pannexin protein channels [10–15]. Of particular note is the Piezo1 channel, which is an essential mechanical sensor involved in many biological processes, especially in bone formation. Piezo1’s intrinsic mechanical sensitivity allows it to function independently of other proteins or second messengers in signal transduction [16–18]. Sun et al. [19] demonstrated that Piezo1 is expressed in both primary osteoblasts and osteocytes, mediating Ca^2+^ influx upon activation and regulating osteogenesis through downstream Ca^2+^ signaling. Knockout studies of Piezo1 in osteoblast cell lines revealed impaired osteogenic function and severe bone structure and strength deficiencies. Mice lacking the Piezo1 gene exhibited a diminished osteoblastic response to mechanical forces and weightlessness. These findings suggest that Piezo1 plays a crucial role in treating osteoporosis or severe bone loss due to mechanical unloading. Bera et al. [20] also reported that TRPV4 is activated by increased extracellular fluid viscosity, which mediates Ca^2+^ influx to enhance RhoA-dependent cellular contractility, ultimately promoting cell migration and tumor metastasis. Therefore, MSCs in the cellular microenvironment are important targets for the precise regulation of ontogeny, physiology, and metabolism, among other life processes. However, current strategies for regulating MSCs are primarily limited to 2D systems, such as fluid shear stress or mechanical stretching. For example, He et al. [21] used mechanical stretching devices in vitro to cyclically stretch the Piezo1 ion channel, promoting hypertrophic scar formation, while Trotier et al. [22] exposed cells to milli-level shear stress to regulate Piezo1 activity. Despite these advances, a remote-controlled strategy in 3D environments is highly sought after for tissue engineering applications. Therefore, there is an urgent need to design in situ, noninvasive, biomimetic systems to precisely regulate MSCs and control cellular physiological functions.

Responsive hydrogels are highly promising materials for creating 3D dynamic environments. They have been extensively applied in various biomedical fields, including wound healing, tissue regeneration, and drug delivery [23,24]. However, most hydrogels lack the fibrous structure and nonlinear mechanical behavior that are characteristic of the natural ECM, which are essential for cell–matrix interactions. A class of biomimetic hydrogels, polyisocyanopeptide (PIC), demonstrates collagen-like fibrous structure and nonlinear mechanics, known as strain-stiffening effect [25]. This means that the stiffness of the PIC hydrogel increases in response to external or cellular stress, playing a critical role in regulating cell function, and facilitating intercellular mechanical communication [26]. Our previous work has shown that PIC can respond to cellular force forming bidirectional cell–matrix interactions, such as cell-induced fiber densification and alignment, and long-range cell–cell communication in 3D, demonstrating their highly biomimetic nature and advantages in cell–matrix interaction manipulation [27]. In addition, PIC hydrogels are thermal responsive. Several single polymers assemble into bundle structures through hydrophobic interaction upon warming up, driven by the oligo-ethylene glycol (OEG) tail at their side chain [28].

Conjugated polymers (CPs), known for their remarkable optical properties, are being utilized in a wide range of emerging fields, including chemical and biomolecular detection [29,30], bioimaging [31,32], biosensing [33,34], disease diagnosis and treatment [35–37], and photosynthesis [38]. However, the poor water solubility of most CPs has limited their broader use in biomedical applications. To enhance their biocompatibility, researchers have developed multifunctional CP nanoparticles (NPs) by using CPs as the core for biomedical applications. NPs offer several advantages, including high fluorescence quantum yield, excellent photostability, good biocompatibility, low cytotoxicity, and ease of modification, making them highly beneficial for disease diagnosis and treatment [39–43]. In fact, precise remote regulation of various ion channels, such as temperature-sensitive and reactive oxygen species (ROS)-sensitive ion channels, has been successfully achieved using NPs [44,45]. Our group has achieved step-by-step targeting and precise regulation of temperature-sensitive ion channels (TRPV1, TRPA1), ROS-sensitive ion channels (TRPM2), and even ion channels on intracellular organelles based on NPs [44,46–48].

**Fig. 1.**
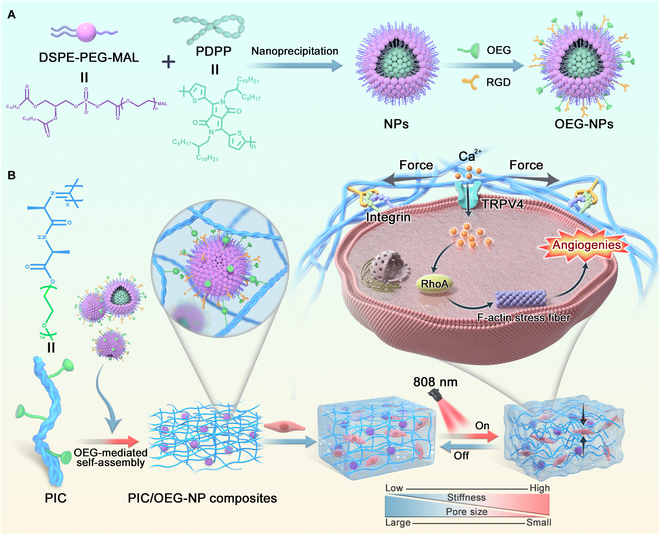
(A) Fabrication of OEG-NPs. (B) Schematic diagram of OEG-mediated assembly of PIC/OEG-NPs composites, and NIR-controlled dynamic mechanical changes of PIC/OEG-NPs composites for regulating MSCs and cell behaviors in 3D.

In this study, we developed a fiber hydrogel composite (PIC/OEG-NPs) with near-infrared (NIR)-controlled dynamic mechanical properties to enable remote regulation of intracellular MSCs in situ (as shown in Fig. 1). Using CPs with excellent photothermal conversion capabilities as the core, NPs were synthesized through a nano-co-precipitation method. OEG was then modified onto the surface via a thiol-maleimide reaction, allowing PIC and OEG-NPs to form PIC/OEG-NPs composites through hydrophobic assembly upon heating. This composite demonstrates good biocompatibility and high photothermal conversion efficiency. Under NIR irradiation, the local heat generated by OEG-NPs, due to their temperature-sensitive nature, enhances hydrophobic interactions within the composite, resulting in a tighter and stiffer gel network. After modification with RGD (arginine–glycine–aspartic acid tripeptide), the PIC/OEG-NPs composites were used to control the activity of MSCs using NIR light, leading to changes in intracellular Ca^2+^. Multiple MSCs, including the transfected Piezo1 ion channel in HEK-293T cells and the naturally expressed TRPV4 ion channel in human umbilical vein endothelial cells (HUVECs), were tested, and a real-time Ca^2+^ flush was achieved by switching the NIR light off and on. Additionally, we explored the relevant intracellular mechanical signal transduction pathways and cytoskeletal protein dynamics. Ultimately, the PIC/OEG-NPs composites were applied to promote angiogenesis in HUVECs.

## Materials and Methods

### Materials and instruments

*Piezo1* plasmid (#80925) was purchased from Addgene. TRPV4 antibody (DF8624) was purchased from Affinity Biosciences. GsMTx4 (HY-P1410) and GSK205 (HY-120691A) were purchased from MedChemExpress. RhoA antibody (10749-1-AP) was purchased from Proteintech. Alexa Fluor secondary antibodies were purchased from Thermo Fisher Scientific. Fluorescein isothiocyanate (FITC)-phalloidin (CA1620) was purchased from Beijing Solarbio Science & Technology Co. Ltd. Sijiqingtai bovine serum (13011-8611) was procured from Zhejiang Tianhang Biotechnology Co. Ltd. Tyrpsin solution (BL512A) was purchased from Biosharp. Dulbecco’s modified Eagle’s medium (DMEM) (PM150210) was purchased from Pricella Life Science & Technology Co. Ltd. The following instruments were used: Shimadzu UV-1800 UV−vis spectrophotometer, BD FACSAria SORP cytometer, JEM 1400 transmission electron microscope, Malvern Nano-ZS90, MDL-H-808 laser generator, and FLIR T420 photothermal camera.

### Preparation of OEG-NPs and OEG-NPs-RGD

Poly-5,5′-(2,5-bis(2-octyldodecyl)-3,6-di(thiophen-2-yl)-2,5-dihydropyrrolo[3,4-c]-pyrrole-1,4-dione (PDPP) (0.5 mg) and 1,2-distearoyl-sn-glycero-3-phosphoethanolamine-maleimide (DSPE-PEG-MAL) (10 mg) were dissolved in 1 ml of tetrahydrofuran using ultrasonication for a duration of 30 min. Subsequently, this mixture was added to 9 ml of ultrapure water, followed by an additional 10 min of ultrasonic treatment. The resulting suspension was then stirred in a fuming cupboard for a period of 5 h, with argon gas being continuously pumped in to effectively remove the organic solvent. The solution was dialyzed for 48 h to completely remove the organic solvent. OEG was added to the filtered solution and stirred at room temperature for 6 h. Following this, dialysis was performed for 24 h to eliminate any unreacted OEG. Next, RGD was introduced to the OEG-NPs solution and stirred at room temperature for 24 h. Another dialysis step of 24 h was conducted to remove any unreacted RGD. According to the absorption coefficient, the concentration of PDPP was determined by Shimazu UV-1800 spectrophotometer.

### Rheology

A stress-controlled rheometer (Discovery HR-2, TA Instruments) was employed to evaluate the mechanical properties of the hydrogels. Prior to testing, the sample was placed on the rheometer plate as a chilled solution (maintained on ice) and subsequently heated from 5 °C to the designated temperature for each group at a uniform rate of 1.0 °C/min. In order to ascertain the storage modulus (*G′*), the samples underwent oscillatory deformation. The gelation temperature was defined as the point at which the storage modulus *G′′* began to increase with a rise in temperature. The nonlinear regime was explored by using a prestress protocol. Under this protocol, the rheometer applied a consistent prestress (*σ_0_*) to the sample, accompanied by a small oscillatory stress (*σ* < 0.1 *σ_0_*) on the sample’s surface, with a frequency ranging from *ω* = 10 to 0.1 Hz at the respective heating temperature. The differential modulus (*K′*) was defined as *K′* = *∂σ*/*∂γ*, and its values were plotted as a function of the applied prestress at *ω* = 1.0 Hz.

### Photothermal study

PIC/OEG-NPs mixed solution was subjected to laser irradiation. The temperature of the solution was continuously tracked by a photothermal camera (FLIR T420) until it reached a maximum, which occurred approximately after 5 min. Heating and cooling cycles were repeated 4 times to assess the photothermal stability of PIC/OEG-NPs composites hydrogel.

### Cell culture and transfection

HEK-293T cells were cultivated in DMEM (High Glucose) medium devoid of sodium pyruvate, within a 37 °C incubator maintained at 5% CO_2_. Similarly, HUVECs were grown in RPMI 1640 under the same incubator conditions. One day prior to transfection, the cells were prepared and seeded into the wells. To facilitate transfection, a mixture was prepared containing 2 μg of the Piezo1 plasmid, 6 μl of DNA transfection reagent, and 200 μl of Opti-MEM. This mixture was then allowed to incubate for 30 min to ensure proper complex formation. Following the incubation period, the transfection complex was gently added to the cell culture wells. The effectiveness of the transfection was subsequently evaluated by confocal laser scanning microscopy (CLSM) 24 h post-transfection.

### Cell viability assay

The cell viability of HEK-293T cells cultured in a PIC/OEG-NPs composite hydrogel was assessed using the cell counting kit-8 (CCK-8; Dojindo Molecular Technologies). Specifically, the cells were 3D cultured in the hydrogel with varying concentrations of OEG-NPs for either 1 or 3 d in a 37 °C incubator with 5% CO_2_. Four hours prior to measurement, an appropriate amount of CCK-8 solution was added to each well. The absorbance of the cultures at 450 nm was then measured to calculate cell viability.

### Ca^2+^ fluorescence image

For Ca^2+^ imaging, HEK-293T cells and HUVECs were encapsulated within composite hydrogels for a period of 3 d. Prior to staining, all solutions were preheated to 37 °C. The composite hydrogel was initially washed twice with phosphate-buffered saline (PBS) and then incubated with a Rhod 2-AM working solution, with a volume sufficient to cover the cells. This incubation was carried out at 37 °C for 40 min, followed by the removal of the Rhod 2-AM solution. The cells were then washed twice with PBS to ensure the complete removal of any residual Rhod 2-AM. After washing, PBS was added to cover the cells and incubated for approximately 30 min to allow for complete de-esterification of the AM bodies within the cells. Imaging was performed using a CLSM to acquire fluorescence images. Subsequently, we employed ImageJ software to randomly analyze the average fluorescence intensity of 3 parallel experiments in each group, thereby ensuring that the analyzed data accurately represented the overall experimental outcomes.

### Immunofluorescence analysis

For immunofluorescence analysis, HEK-293T cells and HUVECs were encapsulated within the PIC/OEG-NPs composite hydrogel for a duration of 3 d. Prior to staining, all solutions were preheated to 37 °C. The composite hydrogel was initially washed twice with PBS and then fixed with 4% paraformaldehyde (PFA) for 1 h. The supernatant was removed and then washed twice with PBS. The samples were then permeabilized with 0.1% Triton X-100 for 30 min and blocked with 1% goat serum overnight. Without washing, the composite hydrogel was incubated with RhoA or vascular endothelial growth factor (VEGF) antibody at 37 °C overnight. The next day, the samples were washed twice with PBS, with each wash lasting 1 h. Subsequently, a goat anti-rabbit secondary antibody was added and incubated at 37 °C for 4 h. The cells were then stained with 4′,6-diamidino-2-phenylindole (DAPI) and washed 3 times with PBS. Fluorescence images were acquired using CLSM. For actin staining, HEK-293T cells and HUVECs were encapsulated within PIC/OEG-NPs composite hydrogel for 3 d. The composite hydrogel underwent 2 washes with PBS and was subsequently fixed with 4% PFA for a duration of 1 h. The supernatant was removed and then washed twice with PBS. Phalloidin was subsequently added and allowed to incubate at 37 °C. Following this, the cells were stained with DAPI and then washed thoroughly 3 times with PBS, with each wash lasting for 5 min. Imaging was conducted once again using a confocal laser scanning microscope for the acquisition of fluorescence images. Subsequently, we employed ImageJ software to randomly analyze the average fluorescence intensity of 3 parallel experiments in each group, thereby ensuring that the analyzed data accurately represented the overall experimental outcomes.

### Angiogenesis experiment

The day preceding the experiment, Matrigel was placed in an icebox and refrigerated at 4 °C to ensure a gradual overnight thaw. On the day of the experiment, a μ-Slide Angiogenesis slide was utilized, with 10 μl of Matrigel added to each well. The slide was then placed in an incubator for approximately 30 min to allow the Matrigel to solidify. Meanwhile, preparations for the cell suspension were initiated. Three days before conducting the angiogenesis experiment, HUVECs were encapsulated within a composite hydrogel for a period of 3 d. On the day of the experiment, leveraging the reversible thermal response of PIC hydrogel, the sample transformed into a liquid state after being placed on ice for 5 min. Subsequently, the cells were harvested via centrifugation. The density of the cell suspension was adjusted to 2 × 10^5^ cells per ml. After thorough mixing, the cell suspension was dispensed into each well of the Matrigel angiogenesis slide. After placing in the incubator, the images were collected periodically according to the growth rate of the cells, and the total tube length and number of branches were meticulously measured and recorded, with subsequent statistical analysis conducted.

### Statistical analysis

The data were presented in the format of mean ± standard deviation (SD). For statistical comparisons between 2 groups, Student’s *t* test was employed. When evaluating statistical comparisons involving more than 3 groups, a one-way analysis of variance (ANOVA) was employed. Statistical significance was indicated by *P* values, specifically denoted as follows: *P < 0.05, **P < 0.01, ***P < 0.001. All statistical analyses were performed using Excel software.

## Results and Discussion

### Preparation and characterization of PIC/OEG-NPs composites

**Fig. 2.**
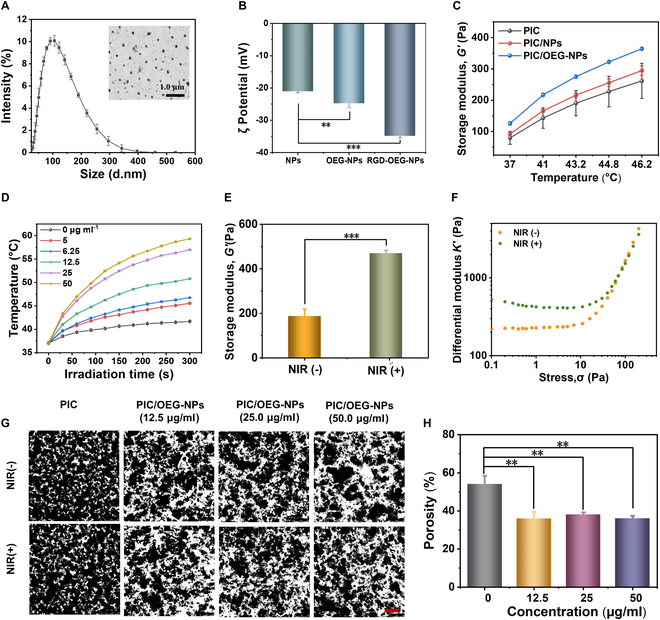
(A) Particle size measurement and representative TEM images of OEG-NPs. (B) ζ potentials for OEG-NPs, OEG-NPs, and OEG-NPs-RGD in PBS. The data are exhibited in the form of mean ± SD, *n* = 3, **P < 0.01, ***P < 0.001 (Student’s *t* test). (C) Storage moduli *G*′ as a function with respect to the temperature of PIC hydrogels, PIC/OEG-NPs composite hydrogels, and PIC/OEG-NPs composite hydrogels heated from 37 to 46.2 °C. (D) Temperature profiles of PIC/OEG-NPs composites at different concentrations under NIR light irradiation (808 nm, 0.8 W cm^−2^). Note: Error bars are present in the figure, but their small size makes them less distinguishable. (E) Storage moduli *G*′ of PIC/OEG-NPs composites with or without NIR light irradiation. Note: Due to the inability to observe the effect of NIR on rheology in real time, the modulus of the sample was measured at the same temperature based on the temperature measurements. (F) Differential modulus (*K*′) against stress σ for PIC hydrogel and PIC/OEG-NPs composites. (G) Confocal images of PIC hydrogel and PIC/OEG-NPs composite hydrogel with or without NIR light irradiation (0.8 W cm^−2^). (H) Statistical analysis of hydrogel porosity. The data are exhibited in the form of mean ± SD, *n* = 3, **P < 0.01 (Student’s *t* test).

The NPs were initially self-assembled from PDPP and DSPE-PEG2000-MAL via nanoprecipitation. Dynamic light scattering (DLS) measurements revealed that the average diameter of the NPs was approximately 100 nm, while transmission electron microscopy (TEM) confirmed good dispersion and a spherical morphology (Fig. 2A, inset, and Fig. S1 with a larger magnification). To achieve the assembly between PIC and NPs, we modified the OEG side chain on the NPs surface using a thiol-maleimide reaction. Rheological analysis showed that PIC/OEG-NPs composites exhibited much higher storage modulus at equivalent temperatures compared to samples without OEG grafting (Fig. 2C), indicating that the OEG side chains actively mediate assembly between PIC and NPs, further enhancing the composites’ mechanical properties. Encapsulation of CP PDPP within NPs endows the PIC/OEG-NPs composites with excellent photothermal conversion capabilities. As shown in Fig. 2D, under 808-nm laser irradiation at a fluence rate of 0.8 W cm^−2^, the temperature peak rose with the increase of both irradiation time and OEG-NPs concentration. After 4 cycles of heating and natural cooling, no reduction in the heating ability of OEG-NPs was observed, indicating excellent photothermal stability (Fig. S2C). Infrared thermal imaging revealed that the brightness of NPs gradually increased with prolonged laser irradiation (Fig. S2D). Additionally, the photothermal conversion efficiency of OEG-NPs was calculated to be 53.12% (Fig. S2E and F).

Meanwhile, PIC demonstrated excellent temperature sensitivity, with its storage modulus gradually increasing as temperature rose. Stress-controlled rheometry confirmed that NIR light effectively regulated the mechanical properties of PIC/OEG-NPs composites. Under NIR irradiation, the storage modulus of PIC/OEG-NPs composites reached 470 Pa, markedly higher than the 187 Pa observed without irradiation (Fig. 2E). In addition, the volume of the PIC/OEG-NPs composites exhibited no marked shrinkage or swelling before and after laser irradiation, indicating that it would not inflict damage on the tissue for applications in vivo (Fig.S3). Additionally, the storage modulus of PIC/OEG-NPs composites increased with rising NPs concentration; however, at very high concentrations, the modulus decreased (Fig. S4A and B). Analyzing particle size changes and scanning electron microscopy (SEM) images of the NPs before and after heating at corresponding concentrations (Figs. S4C and D and S5), we speculated that an excessive amount of NPs might interfere with PIC fiber bundle formation, thereby resulting in a decrease in the storage modulus of the composite hydrogels. To address this, we optimized the conditions and selected 1 mg/ml PIC and 12.5 μg/ml OEG-NPs for subsequent cell biology experiments.

Furthermore, the morphology of PIC/OEG-NPs composites was further characterized using CLSM and SEM. The results showed that the assembly of OEG-NPs enhanced crosslinking within the PIC network, forming larger bundles and reducing pore sizes. Upon NIR irradiation, porosity decreased further, aligning with the rheological results (Fig. 2G and H). Notably, the PIC/OEG-NPs composites retained stress-stiffening properties, with an increase in the modulus index. Due to the temperature increase under NIR light, the storage modulus also rose, leading to a higher plateau modulus (*G*₀). Beyond critical stress, the differential modulus (*K′*) increases exponentially (Fig. 2F), indicating that PIC/OEG-NPs composites exhibit excellent ECM-like nonlinear mechanical properties.

### Dynamically controlling the activity of both Piezo 1 in transfected HEK-293T cells and endogenous TRPV4 in HUVECs

**Fig. 3.**
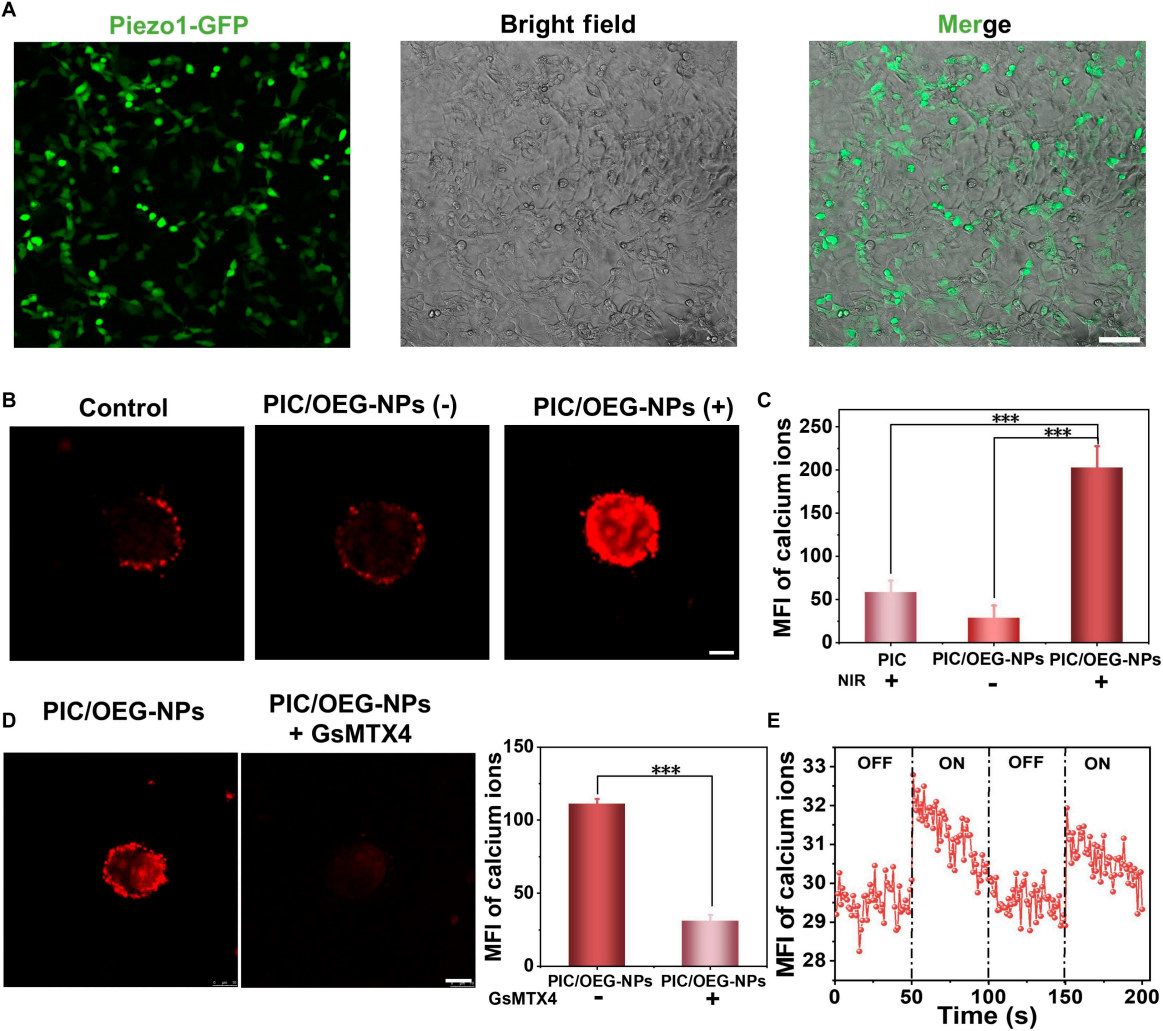
(A) Images showing the expression of Piezo1-GFP (green fluorescent protein) in transfected HEK-293T cells. Scale bar, 100 μm. (B) Rhod 2-AM fluorescent images of HEK-293T cells showing the change of the intracellular Ca^2+^ concentration under different conditions: (1) control group (without OEG-NPs but under NIR irradiation), (2) PIC/OEG-NPs (−) group (with OEG-NPs but without NIR), and (3) PIC/OEG-NPs (+) group (with OEG-NPs under NIR irradiation). Scale bar, 10 μm. (C) Statistical analysis of mean fluorescence intensity. The data are presented in the format of mean ± SD for clear and concise representation, *n* = 3, ***P < 0.001 (Student’s *t* test). (D) Rhod 2-AM fluorescence images of HEK-293T cells reveal changes in intracellular Ca^2+^ concentration with or without Piezo1 ion channel-specific inhibitor. On the right is the statistical analysis of mean fluorescence intensity. Scale bar, 10 μm. The data are presented in the format of mean ± SD for clear and concise representation, *n* = 3, ***P < 0.001 (Student’s *t* test). (E) Changes in the Ca^2+^ mean fluorescence intensity of Piezo1 with the laser irradiation at 808-nm laser switching on/off at an interval of 50 s.

MSCs convert mechanical signals into bioelectric signals in response to mechanical stimuli, integrating these signals within cells and influencing various physiological and pathological processes. Building on these properties, we leveraged the NIR-controlled dynamic mechanical changes of PIC/OEG-NPs composites to regulate MSC activity. First, we characterized the viability of HUVECs within hydrogels of varying stiffness and incubation durations (Fig. S6). After incubating HUVECs within the PIC hydrogel for 5 d, all 3 experimental groups exhibited excellent cell viability. On days 3 and 5, PIC/OEG-NPs composites with NIR irradiation promoted the HUVEC proliferation compared with PIC alone or PIC/OEG-NPs without NIR, indicating good biocompatibility of PIC/OEG-NPs composites (Fig. S6A). Moreover, to investigate the effect of hydrogel stiffness, we assessed cell viability in a stiffer PIC hydrogel (320 Pa, referred to as PIC-2), which was synthesized using a longer polymer length based on our previous work [49]. Although HUVECs continued to proliferate in PIC-2, the proliferation rate was lower than that observed in the softer PIC hydrogel (Fig. S6B). Therefore, the soft PIC hydrogels were chosen as the optimal condition for this study. Next, the cytotoxicity of PIC/OEG-NPs composites was assessed using HEK-293T cells with the CCK-8 assay. As shown in Fig. S7A and B, after being cultured in a 3D manner for 1 or 3 d in composite hydrogels containing varying concentrations of OEG-NPs (0 to 25.0 μg/ml), the viability of HEK-293T cells remained above 90%, indicating the composite hydrogel’s excellent biocompatibility. We then transfected HEK-293T cells with the *Piezo1* gene to evaluate the regulatory effects of PIC/OEG-NPs composites on MSCs, specifically Piezo1. Successful transfection was confirmed by the presence of green fluorescence within the cells (Fig. 3A). To determine ion channel activation, we used the Rhod 2-AM probe to monitor intracellular Ca^2+^ concentration changes. The experimental samples were divided into 3 different groups: (a) the first group was the PIC control group, in which cells were cultured within the PIC hydrogel and then exposed to NIR irradiation; (b) the second group was the PIC/OEG-NPs (−) group, where cells were cultured in the PIC/OEG-NPs composite but not subjected to NIR irradiation; (c) the last group was the PIC/OEG-NPs (+) group, in which cells cultured in the PIC/OEG-NPs composite were exposed to NIR irradiation. As shown in Fig. 3B and C, the red fluorescence intensity in the PIC/OEG-NPs composites with irradiation was significantly higher than in both the PIC-only group and the nonirradiated PIC/OEG-NPs composites group, indicating successful ion channel activation. The subsequent reduction in Ca^2+^ concentration upon the addition of the Piezo1-specific inhibitor GsMTX4 confirmed that the activated ion channel was indeed Piezo1 (Fig. 3D). As illustrated in Fig. 3E, average fluorescence intensity markedly increased during irradiation and steadily declined once the laser was switched off. A real-time video in Fig. S8 illustrated the dynamic changes in fluorescence intensity of Ca^2+^ upon laser on/off.

In order to investigate the importance of thermoresponsive behavior of PIC in PIC/OEG-NPs composites, we employed a temperature-insensitive hydrogel [hyaluronic acid (HA)] as a control. As shown in Fig. S9A and B, Piezo1-transfected HEK-293T cells were encapsulated in HA hydrogels with and without OEG-NPs/NIR, and there were no significant differences in fluorescence intensity across all conditions. Although OEG-NPs generated heat under NIR irradiation, HA lacked the ability to transfer this heat into mechanical tension, thereby failing to activate MSCs. Additionally, we conducted 2D experiments on glass substrates. A similar trend was observed. Neither the control group (without OEG-NPs) nor the OEG-NPs groups exhibited significant differences (Fig. S9C and D). These results confirm that neither NIR light alone nor temperature changes can effectively regulate MSC activity, strengthening the conclusion that the mechanical tension generated by the PIC/OEG-NPs composite is the key factor driving ion channel activation.

**Fig. 4.**
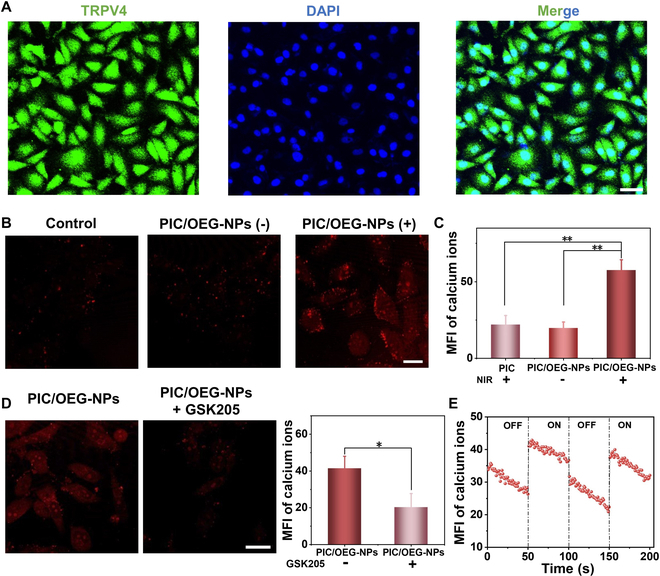
(A) Representative image of immunofluorescence staining of TRPV4 in HUVECs. Scale bar, 50 μm. (B) Rhod 2-AM fluorescent images of HUVECs showing the change of the intracellular Ca^2+^ concentration under different conditions: (1) control group (without OEG-NPs but under NIR Irradiation), (2) PIC/OEG-NPs (−) group (with OEG-NPs but without NIR), and (3) PIC/OEG-NPs (+) group (with OEG-NPs under NIR irradiation). Scale bar, 25 μm. (C) Statistical analysis of mean fluorescence intensity. Data are presented as mean ± SD, *n* = 3, **P < 0.01 (Student’s *t* test). (D) Rhod 2-AM fluorescence images of HUVECs reveal changes in intracellular Ca^2+^ concentration with or without TRPV4 ion channel selective inhibitor. On the right is the statistical analysis of mean fluorescence intensity. Data are presented as mean ± SD, *n* = 3, *P < 0.05 (Student’s *t* test). (E) Changes in the Ca^2+^ mean fluorescence intensity of TRPV4 with the laser irradiation at 808-nm laser switching on/off at an interval of 50 s.

To further evaluate the ability of PIC/OEG-NPs composites to regulate MSCs, we conducted experiments using primary HUVECs, which endogenously express TRPV4. As shown in Fig. 4A, immunofluorescence staining confirmed a high expression of TRPV4 ion channels in HUVECs. Based on previous cell viability findings, it was established that HUVECs maintain high activity in PIC/OEG-NPs composites. Therefore, HUVECs were encapsulated in PIC/OEG-NPs composites for 3 d to allow full cell spreading. Following this, a calcium ion fluorescence probe was applied to track intracellular calcium ion concentration changes, and intracellular fluorescence alterations were observed under NIR irradiation to assess the regulatory effect of PIC/OEG-NPs composites on TRPV4 channels. As seen in Fig. 4B and C, the average fluorescence intensity of the PIC/OEG-NPs composite group increased significantly after irradiation. When a TRPV4-specific inhibitor was added, the red fluorescence intensity was substantially reduced, suggesting the effective activation of the TRPV4 ion channel by PIC/OEG-NPs composites (Fig. 4D). Furthermore, the activation of MSCs within composites assembled with 12.5 μg/ml NPs and PIC hydrogel was considerably more pronounced than that in those with 25.0 μg/ml NPs and PIC hydrogel (Fig. S10), which was consistent with rheological results (Fig. S2A and B). The changes in intracellular mean fluorescence intensity, shown in Fig. 4E, further highlighted the real-time modulation of TRPV4 ion channels by the PIC/OEG-NPs composites. Additionally, we achieved precise remote regulation of Piezo1 ion channel expressed in MC3T3-E1 cells using the PIC/OEG-NPs composites (Fig. S11). Collectively, these results demonstrate that PIC/OEG-NPs composites effectively achieve real-time modulation of the MSCs Piezo1 and TRPV4 upon NIR irradiation.

### Characterization of the mechanosensitive RhoA pathway

**Fig. 5.**
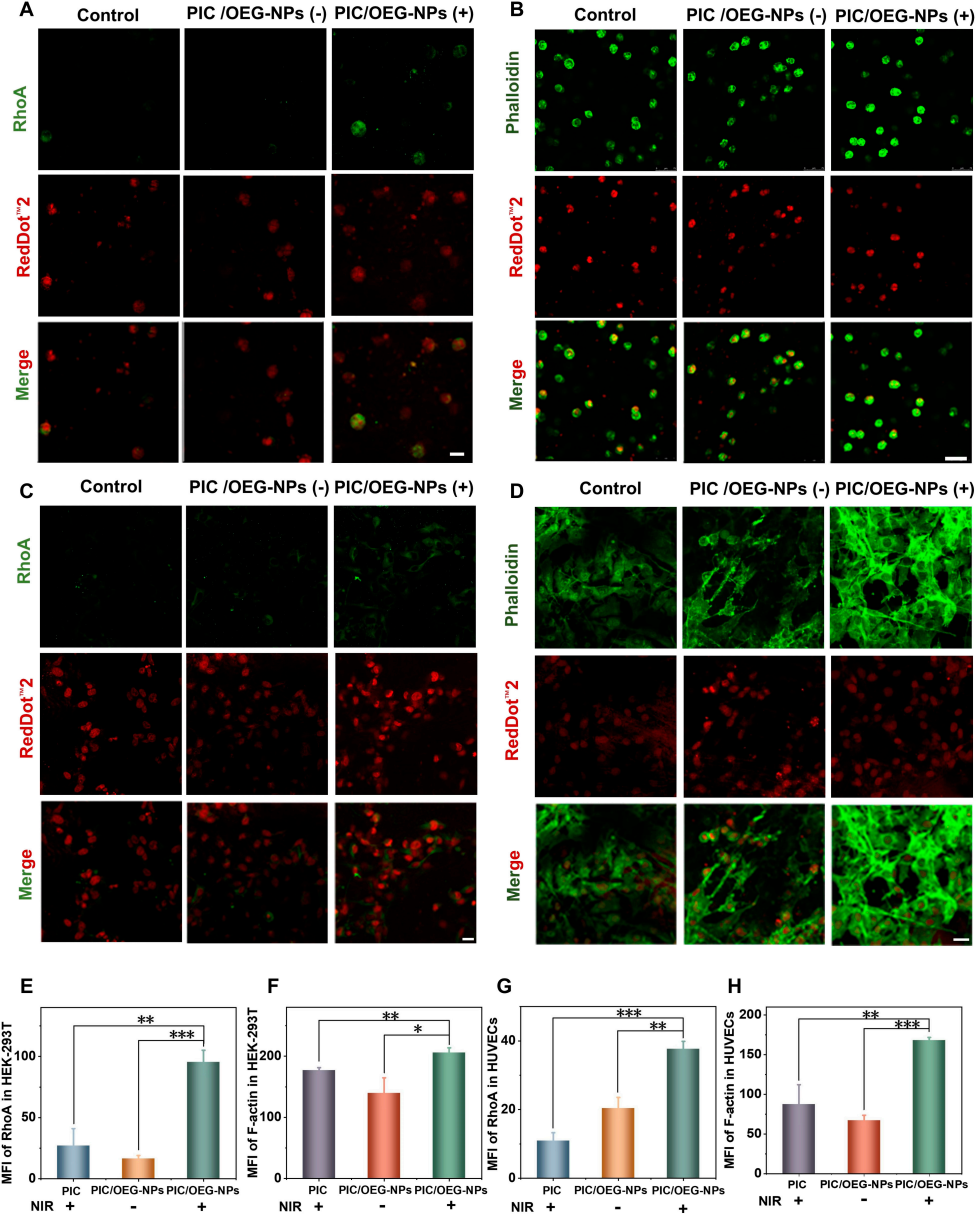
(A) Representative image of immunofluorescence staining of RhoA in HEK-293T cells under different treatment conditions. Scale bar, 25 μm. (B) Representative image of immunofluorescence staining of F-actin in HEK-293T cells under various treatment conditions. Scale bar, 75 μm. (C) Representative image of immunofluorescence staining of RhoA in HUVECs with different treatment conditions. Scale bar, 25 μm. (D) Representative image of immunofluorescence staining of F-actin in HUVECs in different settings of treatment. For (A) to (D), Control represents cells encapsulated in PIC hydrogels without OEG-NPs but under NIR irradiation, PIC/OEG-NPs (−) represents cells encapsulated in PIC/OEG-NPs composites but without NIR, and PIC/OEG-NPs (−) represents cells encapsulated in PIC/OEG-NPs composites under NIR irradiation. Scale bar, 25 μm. (E) Statistical analysis of mean fluorescence intensity of RhoA in HEK-293T cells. Data are presented as mean ± SD, *n* = 3, **P < 0.01, ***P < 0.001 (Student’s *t* test). (F) Statistical analysis of mean fluorescence intensity of F-actin in HEK-293T cells. Data are presented as mean ± SD, *n* = 3, *P < 0.05,**P < 0.01 (Student’s *t* test). (G) Statistical analysis of mean fluorescence intensity of RhoA in HUVECs. Data are presented as mean ± SD, *n* = 3, **P < 0.01, ***P < 0.001 (Student’s *t* test). (H) Statistical analysis of mean fluorescence intensity of F-actin in HUVECs. Data are presented as mean ± SD, *n* = 3, *P < 0.05, **P < 0.01 (Student’s *t* test).

After demonstrating that PIC/OEG-NPs composites have the ability to effectively achieve real-time modulation of the sites to modulate MSCs, we further investigated their impact on intracellular mechanotransduction pathways. RhoA, a pivotal member of the Rho family guanosine triphosphatases (GTPases), functions as a vital signaling molecule in mechanical signal transduction from the extracellular environment and cytoskeletal dynamics. It plays a central role in cytoskeleton reorganization, and notably, this pathway can be activated by calcium influx. To elucidate the effects of PIC/OEG-NPs composites on RhoA expression and its downstream target, F-actin, we conducted experiments in both HEK-293T cells and HUVECs. As illustrated in Fig. 5A and C, RhoA expression significantly increased in the PIC/OEG-NPs composites group following irradiation. Quantitative analysis of the mean fluorescence intensity of RhoA in HEK-293T cells and HUVECs (Fig. 5E and G) revealed that in HEK-293T cells, RhoA expression in the PIC/OEG-NPs (+) group was 3.5 times higher than in the control group and 5.8 times higher than in the PIC/OEG-NPs group without irradiation. Similarly, in HUVECs, RhoA expression in the PIC/OEG-NPs (+) group was 3.5 times higher than in the control group and 1.8 times higher than in the PIC/OEG-NPs (−) group. Given that activated RhoA promotes the formation of actin stress fibers, we next examined the expression levels of F-actin across the different treatment groups after irradiation. As depicted in Fig. 5B and D, F-actin expression was highest in the PIC/OEG-NPs (+) group. Statistical analysis of mean fluorescence intensity (Fig. 5F and H) indicated that in HEK-293T cells, F-actin levels in the PIC/OEG-NPs (+) group were 1.2 times higher than in the control group and 1.5 times higher than in the PIC/OEG-NPs (−) group. In HUVECs, F-actin expression was 1.9 times higher in the PIC/OEG-NPs (+) group compared to the control group, and 2.5 times higher than in the PIC/OEG-NPs (−) group. These results suggest that irradiation of PIC/OEG-NPs composites activates intracellular MSCs, induces extracellular calcium influx, and triggers intracellular signal transduction pathways, thereby resulting in enhanced expression of RhoA and its downstream effector, F-actin.

### NIR-controlled angiogenesis using PIC/OEG-NPs composites

**Fig. 6.**
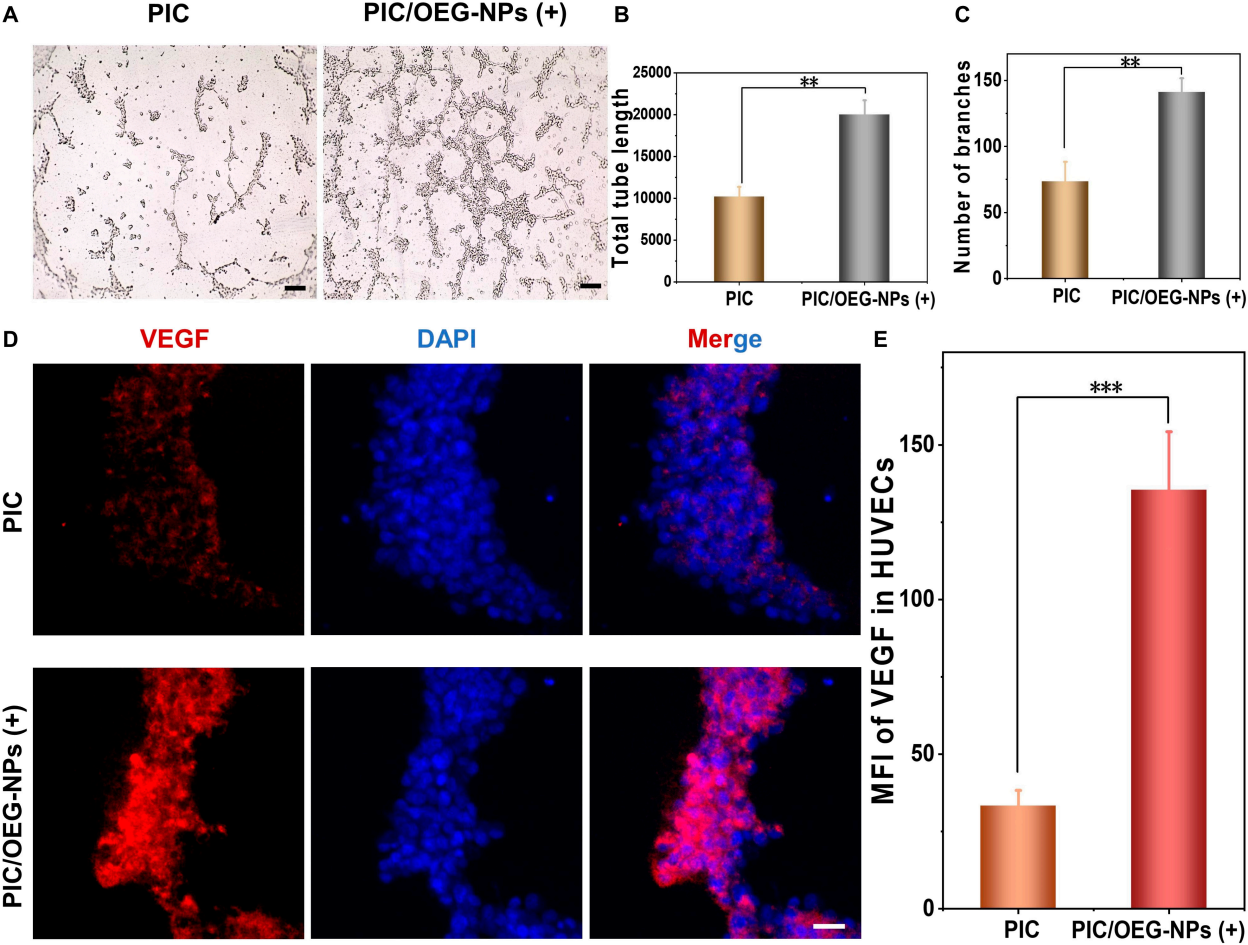
(A) Images of tube formation of HUVECs. Scale bar, 200 μm. (B and C) Quantitative statistics of total tube length and the number of branches. Data are presented as mean ± SD, *n* = 3, **P < 0.01 (Student’s *t* test). (D) Representative image of immunofluorescence staining of VEGF in HUVECs. Scale bar, 50 μm. (E) Statistical analysis of mean fluorescence intensity. Data are presented as mean ± SD, *n* = 3, ***P < 0.001 (Student’s *t* test).

Angiogenesis refers to the intricate process of developing new capillary-like blood vessels that originate from existing capillaries and post-capillary venules. It is regulated by multiple factors. When the ECM is distorted by mechanical forces, cellular mechanical sensors (such as MSCs) are activated, triggering mechanical signaling pathways that promote angiogenesis. TRPV4 ion channels are involved in regulating systemic endothelial cell (EC) functions such as vasodilation and permeability and have been shown to regulate angiogenesis via Ca^2+^ signaling. Previous studies have demonstrated that the expression and assembly of F-actin are crucial in promoting EC migration and angiogenesis. The primary step in angiogenesis is cytoskeleton reorganization, which is crucial for the migration of HUVECs. Therefore, the ability to remotely regulate angiogenesis using NIR light could be highly valuable for advancing tissue regeneration applications. Considering that PIC/OEG-NPs composites can dynamically regulate HUVEC behavior via mechanotransduction pathway, we postulated that this strategy could augment the angiogenic capacity of HUVECs upon NIR irradiation. To verify this hypothesis, we carried out a tube formation assay, which is a commonly employed method for evaluating in vitro angiogenesis due to its strong simulation of in vivo conditions. Prior to the tube formation experiment, HUVECs were cultured in PIC/OEG-NPs composites and exposed to NIR irradiation for 2 consecutive days to induce angiogenic activity. As shown in Fig. 6A, a substantial number of capillary-like structures were observed in the PIC/OEG-NPs group after irradiation. The total tube length and number of branches in the NIR (+) group were significantly increased, with the total tube length being 1.92 times that of the control group and branch count increasing by 96% (Fig. 6B and C). VEGF, a crucial regulator of angiogenesis, facilitates EC proliferation, promotes vessel formation, and enhances vascular permeability. Therefore, we also measured VEGF expression across the different groups. As illustrated in Fig. 6D and E, VEGF expression was significantly elevated by NIR irradiation. These results demonstrate that PIC/OEG-NPs composites enhance cell angiogenesis after NIR irradiation, highlighting their potential for applications in tissue regeneration.

## Conclusion

In this study, we developed NIR responsive dynamic hydrogels (PIC/OEG-NPs) based on CP NPs grafted with OEG (OEG-NPs) and PIC polymers, aiming to achieve remote regulation of intracellular MSCs. During the gelation of PIC, the hydrophobic interactions between the OEG side chains of PIC and OEG-NPs were instrumental in driving their self-assembly. When exposed to NIR laser irradiation, the OEG-NPs exhibited photothermal conversion properties, efficiently converting light energy into heat. This heat-induced effect not only strengthened the hydrophobic interactions between PIC and OEG-NPs but also led to the formation of a denser and more compact fibrous network within the PIC/OEG-NPs composites. As a result, the mechanical properties of the composites were enhanced, endowing them with the ability to dynamically regulate MSCs, including Piezo1 in transfected HEK-293T cells and endogenous TRPV4 in HUVECs, via NIR irradiation. This modulation further enhanced the angiogenic capacity of HUVECs. Consequently, the activity of MSCs, including Piezo1 in transfected HEK-293T cells and endogenous TRPV4 in HUVECs, could be dynamically regulated through NIR irradiation. This modulation enhanced the angiogenic capacity of HUVECs. Additionally, we investigated the effects of PIC/OEG-NPs composites on intracellular mechanical signal transduction pathways. In conclusion, this work proposes an effective strategy that enables remote, in situ, and precise regulation of intracellular MSCs.

This strategy enables the cell to sense and interpret various signals from the external environment by regulating intracellular MSCs, further converting mechanical signals from the extracellular environment into biochemical signals within the cell. As a crucial carrier of information transmission within cells, biochemical signals can trigger a series of intracellular reactions, thus enabling the regulation of a wide range of cellular processes, including cell proliferation, differentiation, and migration. The ability to precisely regulate cellular processes is of vital importance to the fields of tissue engineering and regenerative medicine. Therefore, this strategy has broad application prospects in the fields of tissue engineering and regenerative medicine and is expected to provide effective means for the treatment of related diseases.

## Data Availability

The datasets used and/or analyzed during the current study are available from the corresponding author on reasonable request.
