# Supplementary material for: Near-Infrared Light-Controlled Dynamic Hydrogel for Modulating Mechanosensitive Ion Channels in 3-Dimensional Environment
Source: Biomater Res. 2025 Apr 9;29:0182. doi: 10.34133/bmr.0182 (PMC11979339; doi:10.34133/bmr.0182)
Supplement: Supplementary 1 — Figs. S1 to S11 [file bmr.0182.f1.docx]

Supporting Information for

**Near-infrared light-controlled dynamic hydrogel for modulating mechanosensitive ion channels in three-dimensional culture**

Xiaoning Liu^1^, Zimeng Zhang^2^, Zhanshuo Cao^3^, Hongbo Yuan^2,4*^, Chengfen Xing^1,2*^

^1^ School of Materials Science and Engineering, Hebei University of Technology, Tianjin 300401, China

^2^ Key Laboratory of Molecular Biophysics of Hebei Province, School of Health Sciences and Biomedical Engineering, Hebei University of Technology, Tianjin 300401, China

^3^ School of Chemical Engineering, Hebei University of Technology, Tianjin 300401, China

^4^ Molecular Imaging and Photonics, Chemistry Department, KU Leuven, Celestijnenlaan 200F, 3001 Heverlee, Belgium

* Corresponding author (email: xingc@hebut.edu.cn; hongbo.yuan@kuleuven.be)

**Supplemental Figures:**


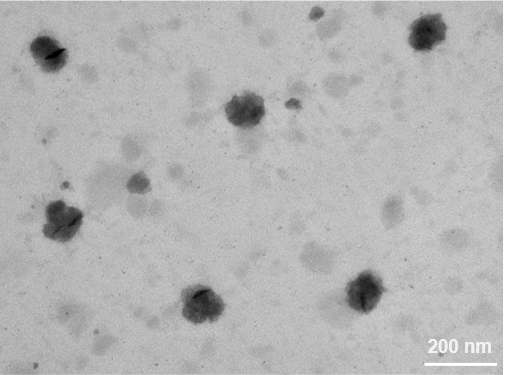


**Fig. S1.** TEM image of OEG-NPs. Scale bar: 200 nm.


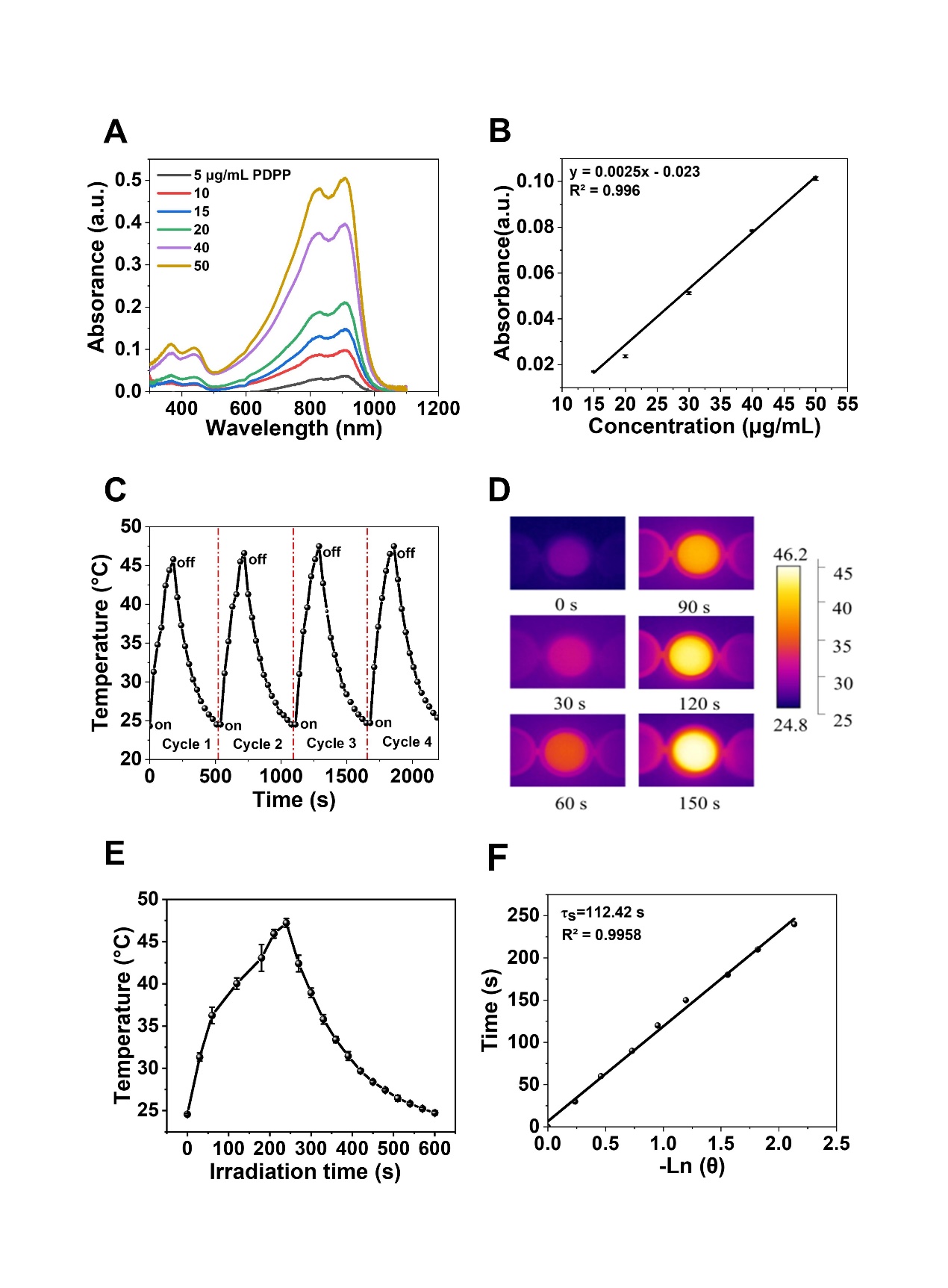


**Fig. S2.** (A) The absorption spectra of different concentrations of PDPP in THF. (B) Extinction coefficient of PDPP. Note: Error bars are present in the figure, but their small size makes them less distinguishable. (C) Stability study of OEG-NPs under the photothermal heating and natural cooling cycles. The concentration of OEG-NPs was 25.0 μg mL^-1^ calculated based on PDPP. (D) Infrared thermal images of the OEG-NPs aqueous solution collected at different laser irradiation times. (E) Temperature elevation of OEG-NPs (25.0 μg mL^-1^) under 808 nm laser irradiation followed by subsequent cooling to room temperature. (F) Time constant for heat transfer is determined to be τ_s_ = 112.42 s by applying the linear time data from the cooling period versus negative natural logarithm of driving force temperature, which is obtained from the cooling stage of (E). The photothermal conversion efficiency was 53.12% for OEG-NPs.


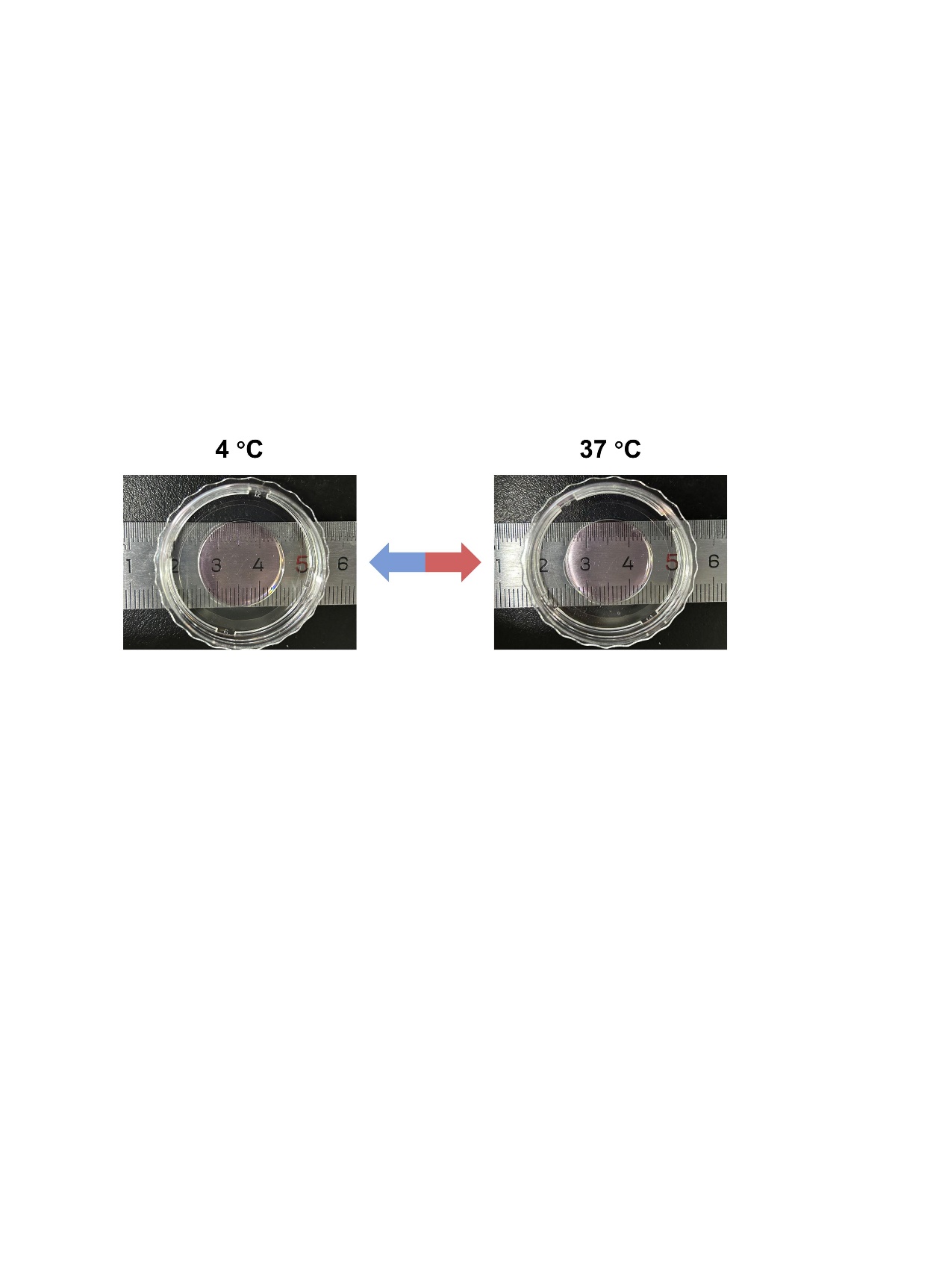


**Fig. S3.** Images of PIC/OEG-NPs composites volume changes during temperature change.


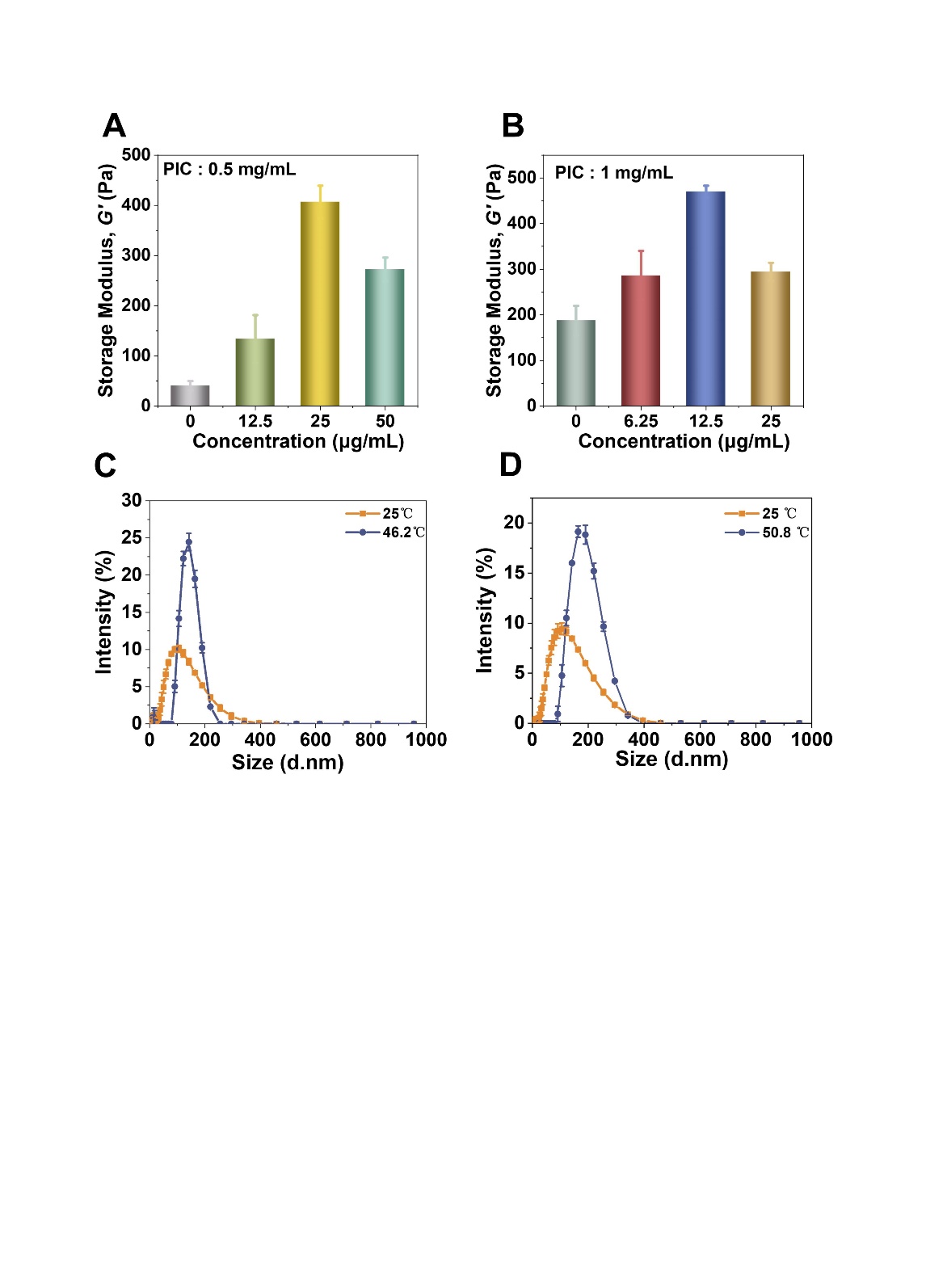


**Fig. S4.** (A) Storage moduli *G′* as a function of temperature of composite hydrogel composed of 0.5 mg mL^-1^ PIC hydrogel and OEG-NPs of different concentrations. (B) Storage moduli G′ as a function of temperature of composite hydrogel composed of 1.0 mg mL^-1^ PIC hydrogel and OEG-NPs of different concentrations. (C) Particle size change of 12.5 μg mL^-1^ nanoparticles before and after irradiation. (D) Particle size change of 25.0 μg mL^-1^ nanoparticles before and after irradiation.


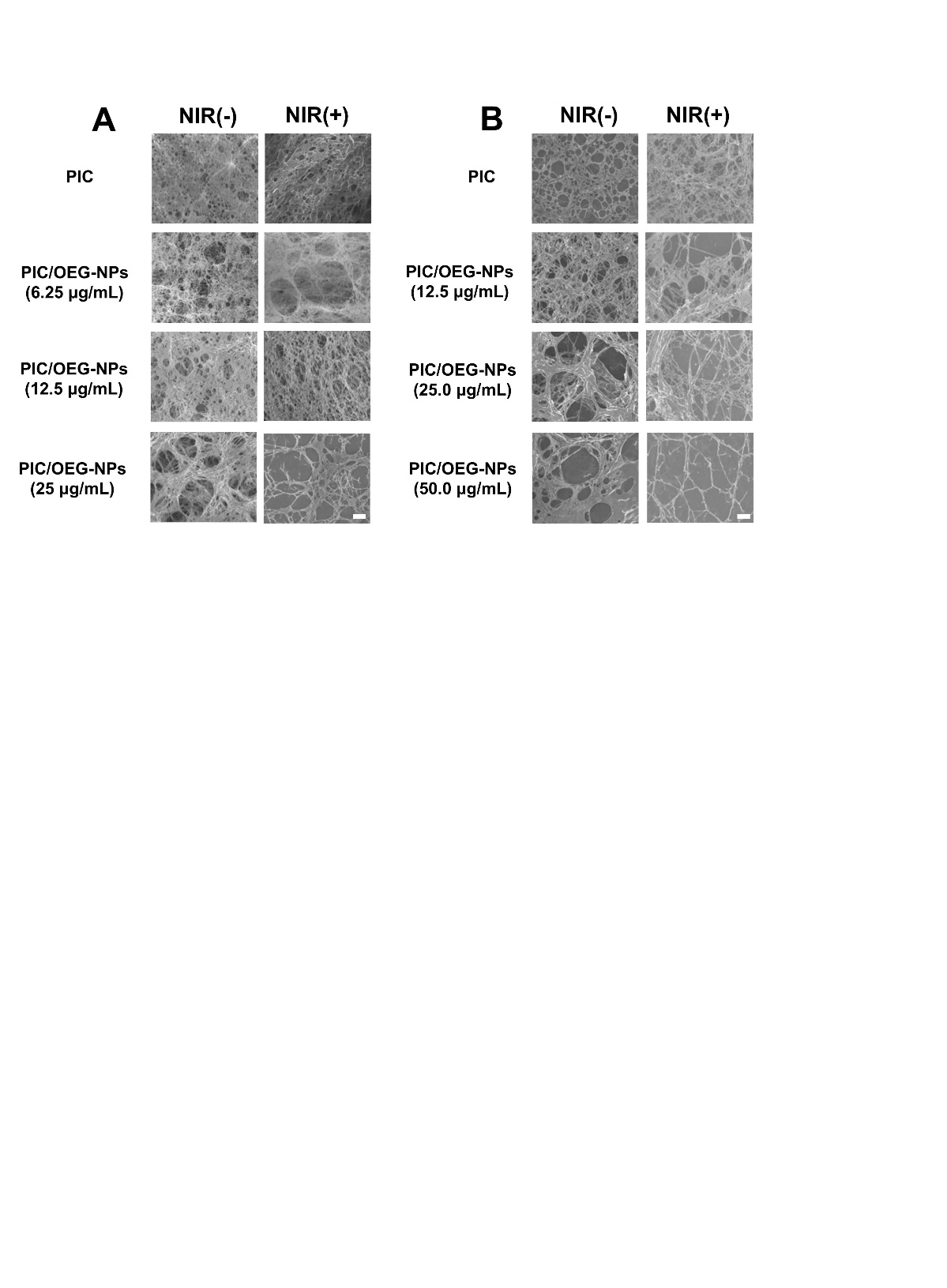


**Fig. S5.** (A) SEM images of composite hydrogel composed of PIC (1 mg mL^-1^) hydrogel and nanoparticles of different concentrations. Scale bar: 10 μm. (B) SEM images of composite hydrogel composed of PIC (0.5 mg mL^-1^) hydrogel and nanoparticles of different concentrations. Scale bar: 10 μm.


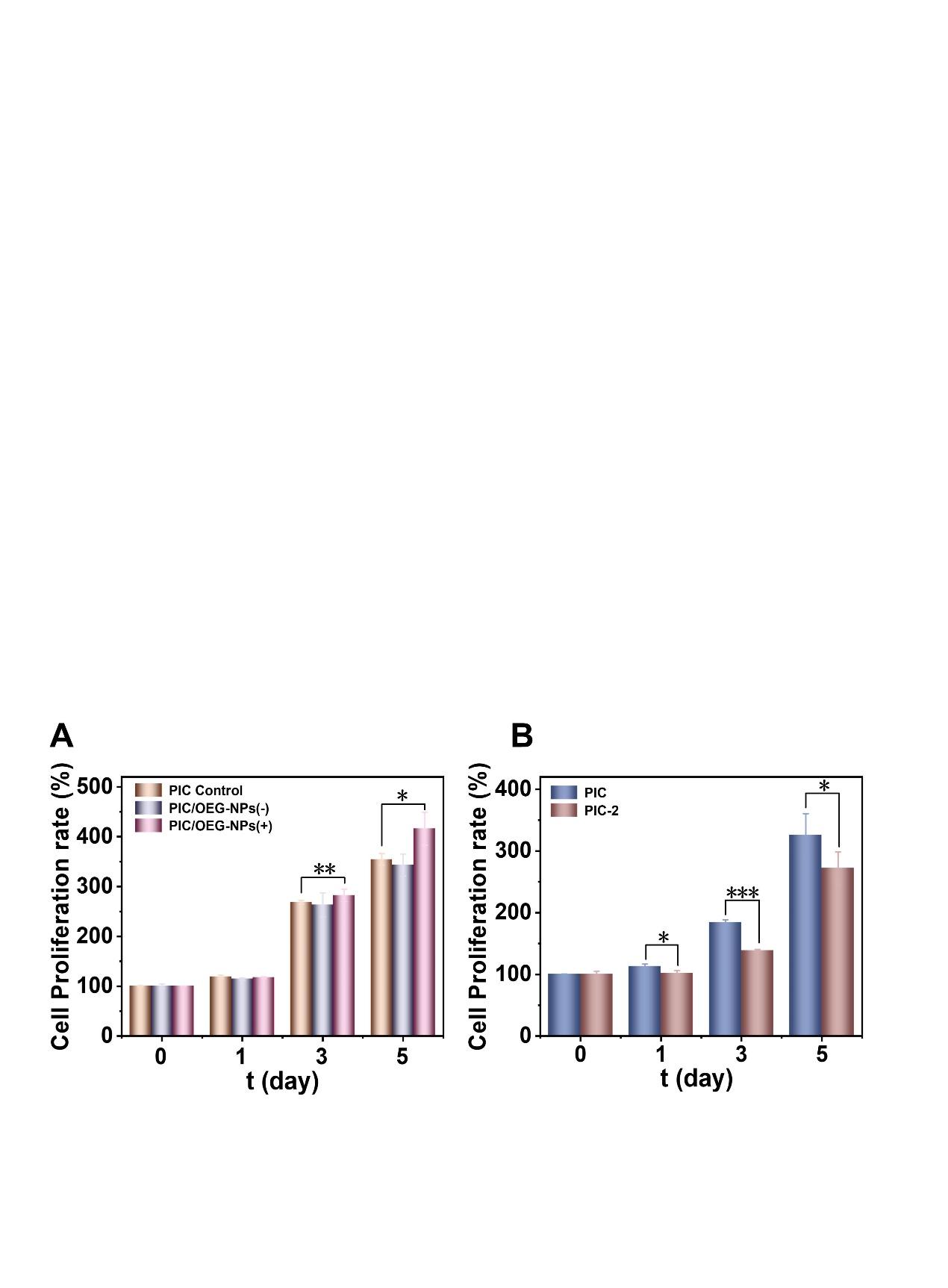


**Fig. S6.** (A) Effect of PIC control group, PIC/OEG-NPs (-) group and PIC/OEG-NPs (+) group on HUVECs proliferation. n=4, **p*＜0.05, ***p*＜0.01 (Student’s t test). (B) Effects of PIC and PIC-2 hydrogels with different stiffnesses on HUVECs proliferation, n=4, **p*＜0.05, ****p*＜0.001 (Student’s t test).


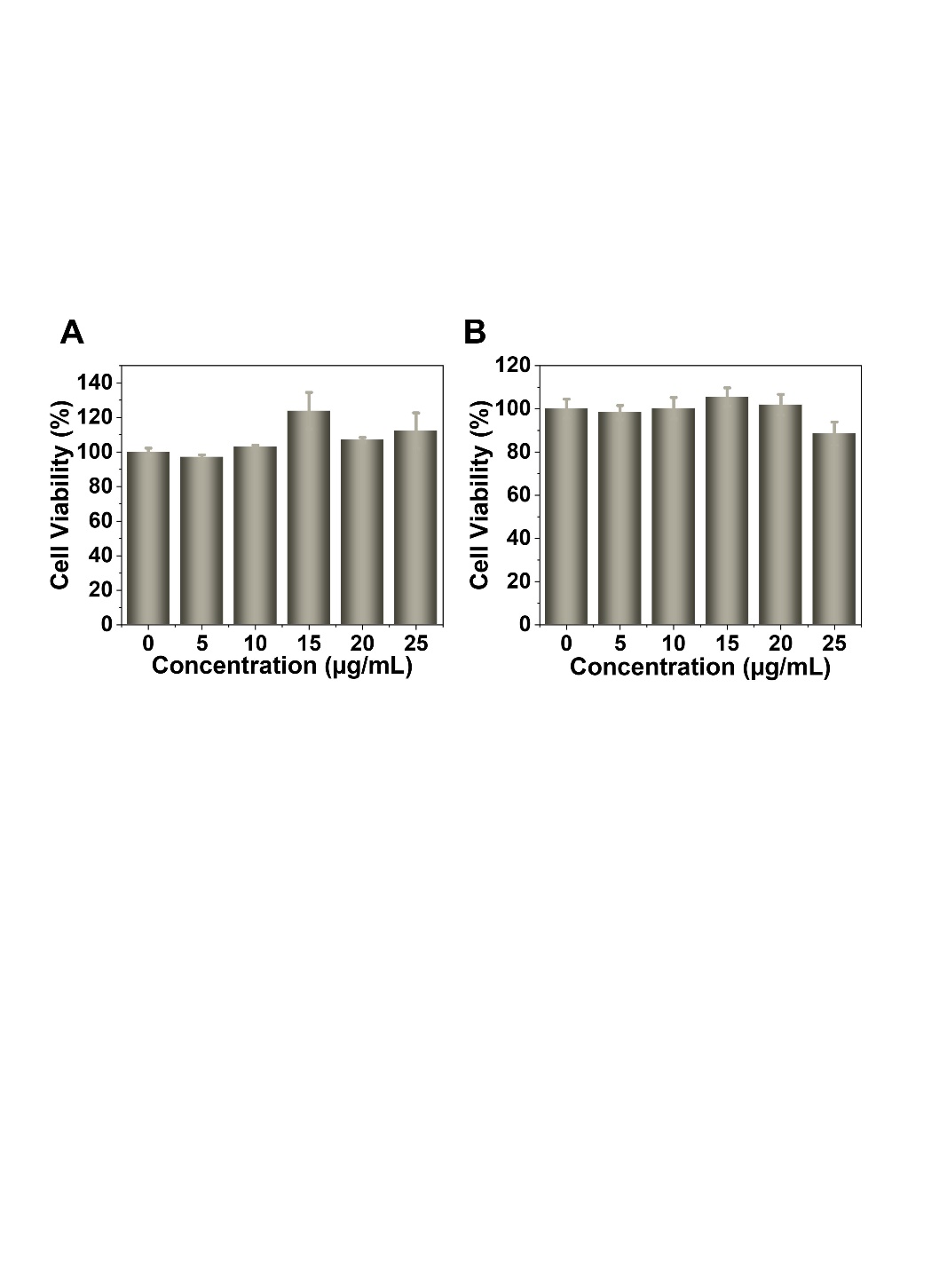


**Fig. S7.** (A) Cell viability of HEK-293T cells cultured with composite hydrogel for 24 hours. (B) Cell viability of HEK-293T cells cultured with composite hydrogel for 72 hours.


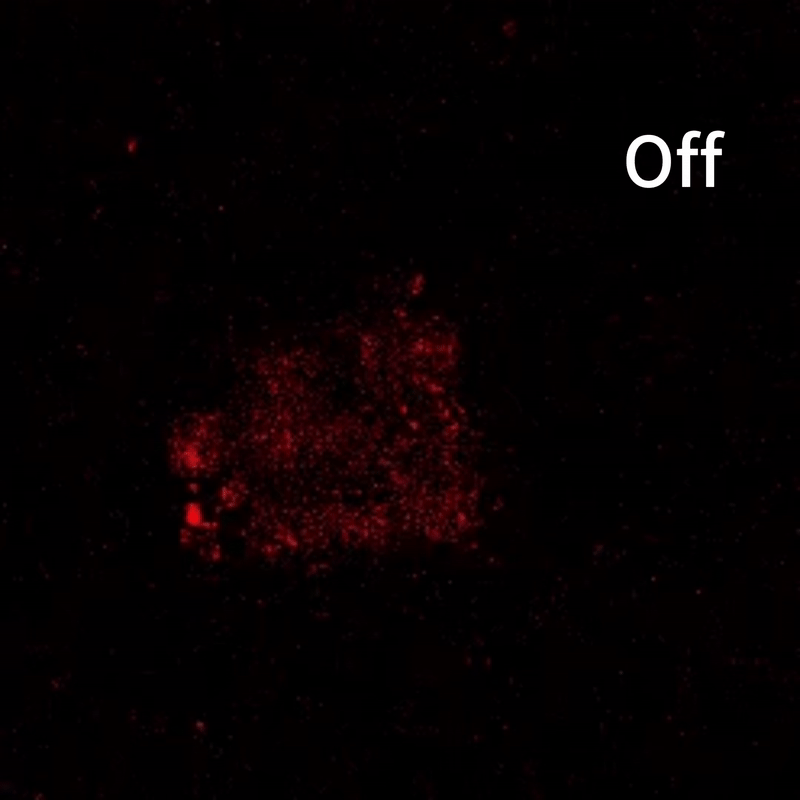


**Fig. S8.** The real-time video of changes in the Ca^2+^ mean fluorescence intensity of Piezo1 with an 808 nm laser irradiation. Scale bar: 30 μm. Note: t=100 s. The laser remains in the off state during the time interval from 0 to 50 s, and then switches to the on state from 50 to 100 s.


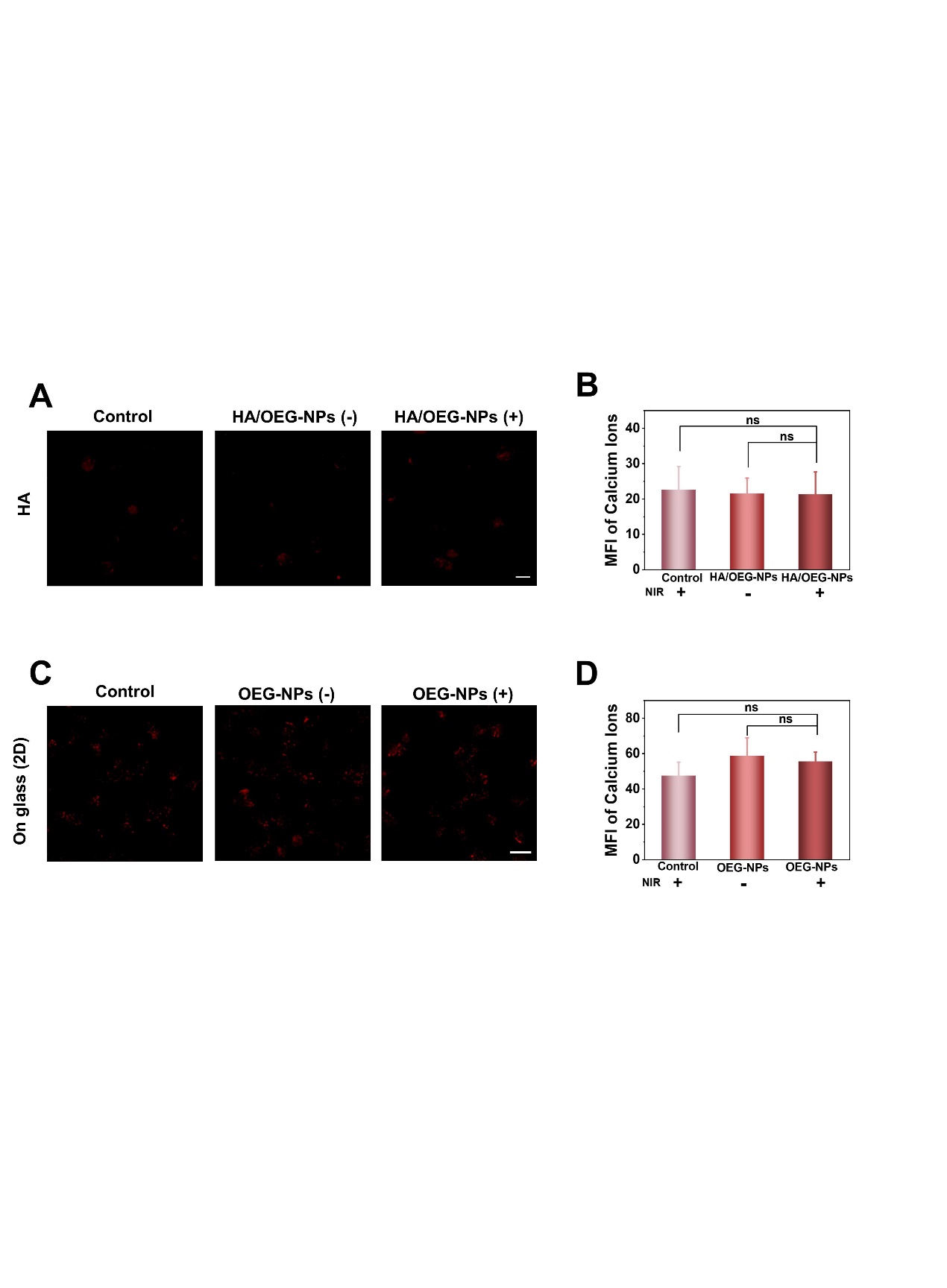


**Fig. S9**. (A) Rhod 2-AM fluorescence images of Piezo 1-transfected HEK-293T cells in the control group (without OEG-NPs but under NIR irradiation), HA/OEG-NPs (-) group (with OEG-NPs but without NIR) and HA/OEG-NPs (+) group (with OEG-NPs under NIR irradiation) in 3D culture. Scale bar: 100 μm. (B) Statistical analysis of mean fluorescence intensity. The data are presented in the format of mean ± SD for clear and concise representation, n=3 (Student’s t test). (C) Rhod 2-AM fluorescence images of Piezo 1-transfected HEK-293T cells in the control group (without OEG-NPs but under NIR irradiation), the OEG-NPs (-) group (without NIR), and the OEG-NPs (+) group (under NIR irradiation) in 2D culture. Scale bar: 30 μm. (D) Statistical analysis of mean fluorescence intensity. The data are presented in the format of mean ± SD for clear and concise representation, n=3 (Student’s t test).


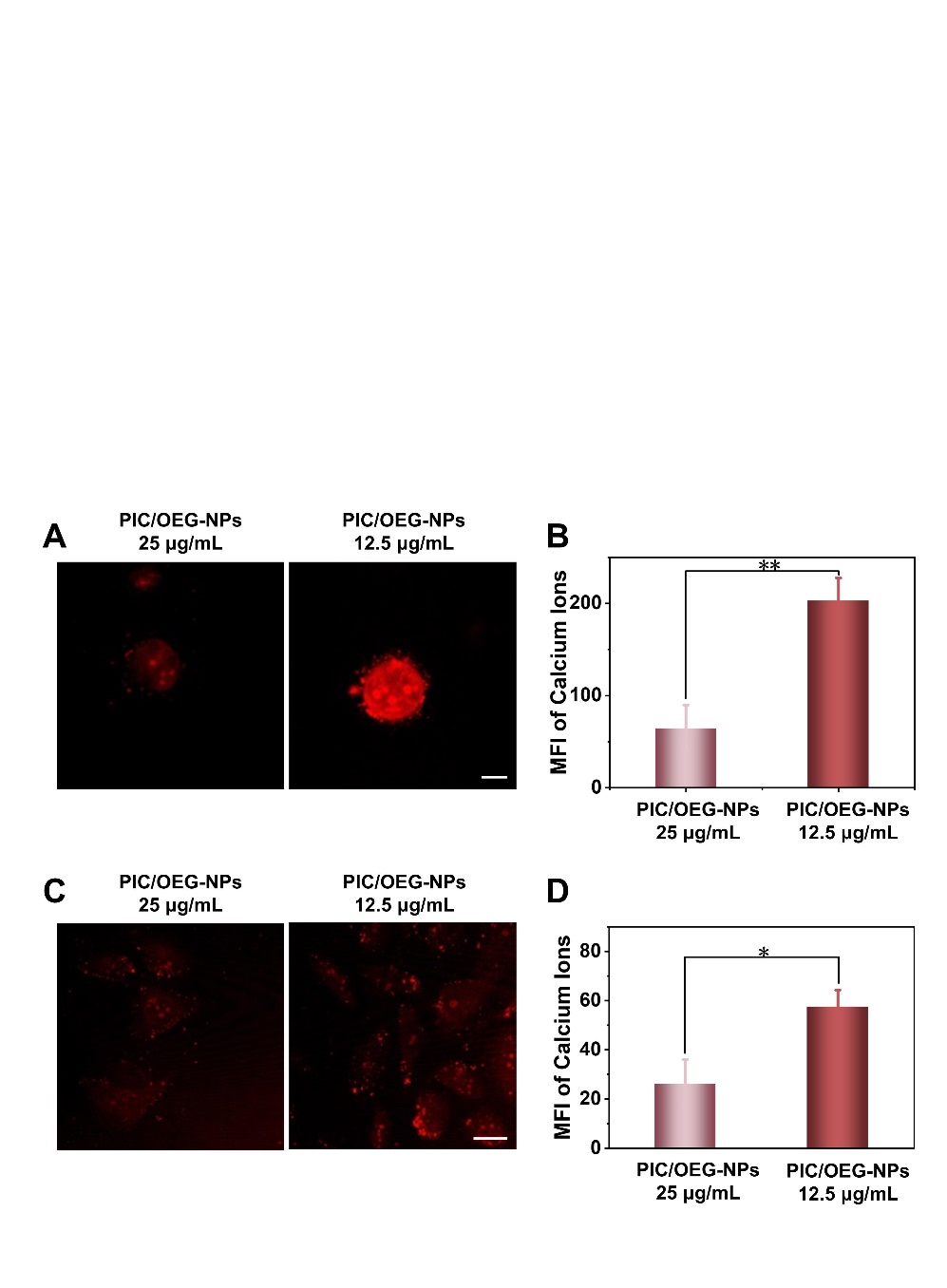


**Fig. S10.** (A) Activation of Piezo1 ion channel by composite hydrogel composed of 12.5 μg mL^-1^ OEG-NPs and 25.0 μg mL^-1^ OEG-NPs and PIC hydrogel. Scale bar: 10 μm. (B) Statistical analysis of mean fluorescence intensity. Data are presented as mean ± SD, n=3.,***p*＜0.01 (Student’s t test). (C) Activation of TRPV4 ion channel by composite hydrogel composed of 12.5 μg mL^-1^ OEG-NPs and 25.0 μg mL^-1^ OEG-NPs and PIC hydrogel. Scale bar: 25 μm. (D) Statistical analysis of mean fluorescence intensity. Data are presented as mean ± SD, n=3.,**p*＜0.05 (Student’s t test).


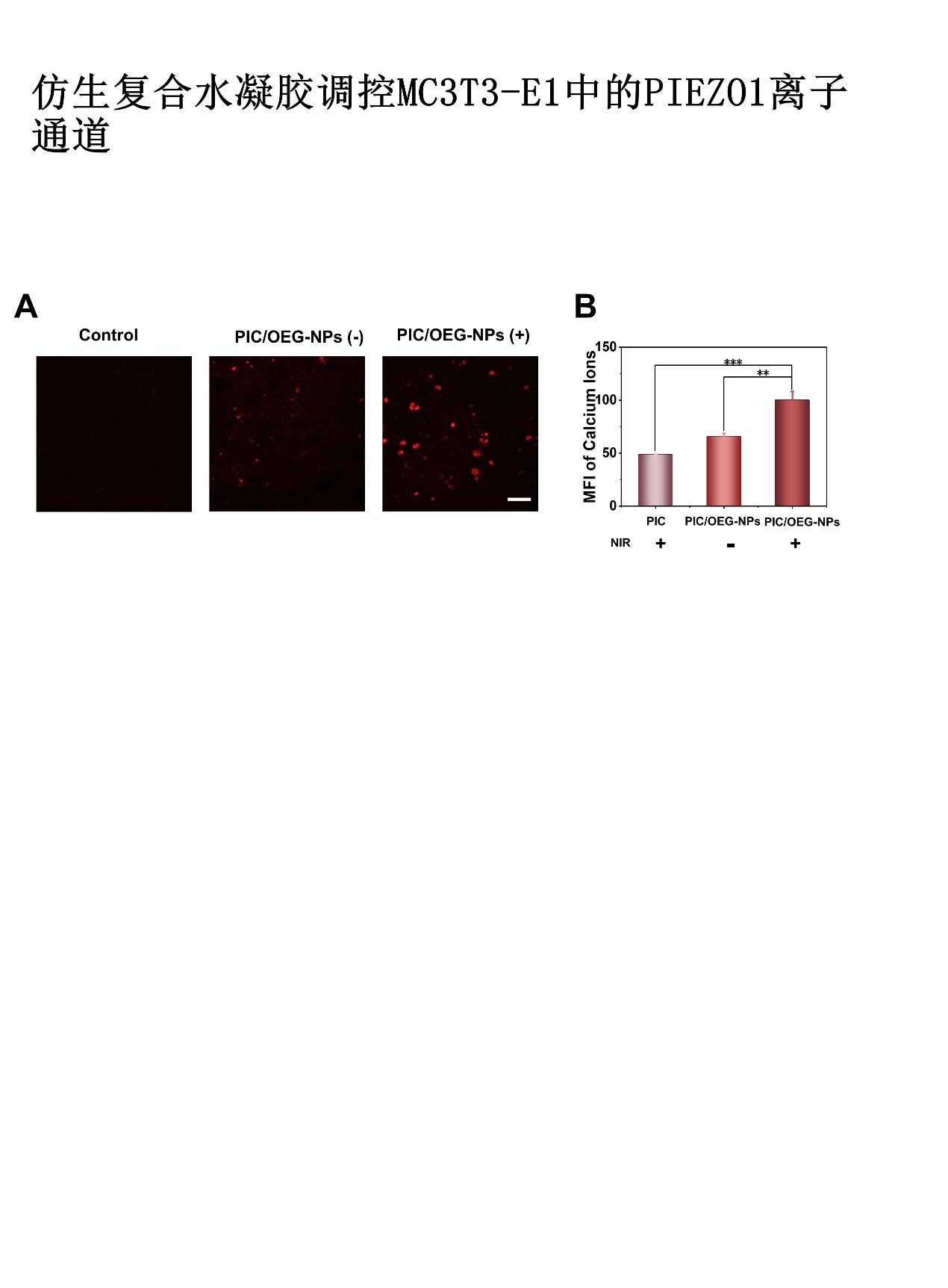


**Fig. S11.** (A) Rhod 2-AM fluorescent images of MC3T3-E1 cells showing the change of the intracellular Ca^2+^ concentration under different conditions. Scale bar: 100 μm. (B) Statistical analysis of mean fluorescence intensity. Data are presented as mean ± SD, n=3.,****p*＜0.001 , ***p*＜0.01 (Student’s t test).
